# Gold Inlays

**Published:** 1908-05-15

**Authors:** Mortimer V. Hopkins


### GOLD INLAYS.

BY DR. MORTIMER V. HOPKINS, D. D. S.

Read before the Michigan First District Society, January 9th, 1908.

In a discussion of the gold inlay problem, it might be well to first consider the questions: Why is the gold inlay needed? What materials and methods is it intended to supplant? What advantages has it to offer not possessed by long established methods?

The skill of the dentist of the past was judged largely by his ability to insert a good, well-condensed gold filling, bringing it out to proper contour, having the margins perfect, and the surface well polished and free from pits and inequalities.

In the training of the dental student the closest attention was given to this subject, and it is not to be wondered at that many of the fillings inserted 25 years or more ago are still doing service. But it is probable that then, as now, a great majority of the gold fillings were less enduring than should be expected of an operation involving such an outlay of labor, time, pain and expense.

The average life of a gold filling is said to be five years. This is only a guess, as it would be impossible to collect evidence which would give us accurate knowledge on this point. It is true, however, that many gold fillings are defective on the very day of their insertion, and sometimes when a filling seems to be free from defects, recurrent caries appear within a year or so thereafter, which necessitates further attention.

If the results obtained by filling cavities with gold foil are approximately as uncertain as stated in the hands of the average operator, it is clearly our duty to investigate any method which promises an improvement.

The development of the porcelain inlay, and the results that have obtained from its use would indicate that the cement inserted filling offers many advantages not possessed by metal fillings, inserted without the aid of cement.

The cement effectually seals the cavity, and a filling so inserted is insured of a reasonable amount of service, even when it possesses defects which would render a gold or amalgam filling useless. The cement acts also, to a certain extent, as a non-conductor of thermal changes, a decided advantage in many cases. Porcelain possesses the fatal defect of friability, and should not be used in shallow cavities which are so situated that great stress is likely to be brought against the filling.

Porcelain inlays are sometimes crushed to pieces by the force of the occluding teeth biting against them. In cases of this kind the gold inlay is clearly indicated.

Defective and broken down molar and bicuspid teeth are often crowned with gold when a gold inlay would give much better results. This statement is often confirmed by advocates of oral prophylaxis, as it is found to be very difficult indeed to properly cleanse the necks and roots of the teeth which have been crowned by banded root crowns.

Amalgam fillings are frequently resorted to, not necessarily because the patient objects to a better or more expensive means of restoring the defective tooth, but because the patient has not the strength to endure the strain and fatigue of one or more hours of the rubber dam, clamps, wedges, etc., which are associated with a gold-foil operation. And in addition, it must be acknowledged, the average operator is doubtful of his ability to insert a successful gold filling in some of the cavities we are called upon to treat.

It would be folly to assert that gold foil and amalgam are to be discarded, and that the gold and porcelain inlays are to be substituted in all cases. These materials have done, and are doing good service, and could not well be dispensed with.

The claim for the inlay is, not that it is to become the universal filling material, but that in many cases which have been treated in the old way the inlay is an improvement.

The gold inlay is stronger than the gold filling because it is melted into shape. It cannot flake nor become pitted on the surface. Leaky gold fillings and the resulting discoloration of the tooth are abominations which are never seen in connection with the gold inlay.

The pain and discomfort which the patient suffers during the insertion of a large gold filling in a lower molar, for instance, is far greater than is experienced when the inlay method is used, and incidentally, the operator is also benefitted, as the work is less exhausting and admits of frequent change of position.

During recent times so much has been written on this subject that it seems impossible to present any original method of inlay construction.

Some of the descriptions seem impractical because of the length of time it takes to complete an inlay, and others seem too complicated to ever come into general use.

My first attempt at inlay work was a modification of a method described at college 18 years ago. The tooth to be filled was an upper bicuspid, with cavities in both the mesial and distal surfaces, decay extending along the fissure uniting both cavities. In this case the decay was removed, the margins cut well back and the fissure considerably enlarged. The entire cavity was then filled with cement. After the cement had hardened it was trimmed slightly at the cavity margins, so that in burnishing the matrix the margins would be clearly defined. The remainder of the cement filling was trimmed away just enough so that a matrix covering the entire cavity might be withdrawn without becoming distorted, and the fissure deepened slightly more than the rest of the cavity. A piece of pure gold of 36 gauge, and approximate shape and size was bent so as to entirely cover the cavity. This was pressed and burnished to place, being removed frequently and trimmed with shears, as the outline of the cavity became marked on the gold by the burnishing process. A piece of platinum wire of suitable size was placed in the matrix along the fissure, and bent over into each cavity;this was soldered to place, and the burnishing completed. The matrix was removed, the reverse side painted with whiting and water, the matrix held with locking tweezers and filled to proper shape and fullness with solder melted by the blow-pipe flame. As much of the finishing as possible was done before cementing to place, and the final finishing being done in the mouth. Inlays of this kind are often of great service, and can be made in less time than is required by some of the other methods.

Another method which has been satisfactory with me in cases of large cavities in the buccal surfaces of lower molars in which decay extends to or slightly under the gum line, may be described as follows: The decay is removed and the margins trimmed back to sound tooth structure. The cavity is then filled with cement. When the cement has hardened enough it is trimmed away to allow for about 28 gauge material. A piece of 1-1000 inlay platinum of suitable size and shape is burnished to place. Platinum is then removed and the reverse side is painted with whiting and water, and enough pure gold flowed into matrix to build out whatever fullness is desired. After being dipped in hydrochloric acid to remove the whiting, the piece is returned to the tooth and a small hole drilled through the inlay and into the cement a short distance from the cavity margin, at each end of the inlay. Platinum pins from a plate tooth are fitted into these holes and soldered. It is an advantage to leave these holes small enough so that the pins will be held in position by the closeness of the fit before soldering. The inlay is now partially finished and cemented to place, the final finishing being done in the mouth.

In cases in which a sound tooth has been fractured by accident, and it does not seem advisable to restore the broken portion with porcelain, a gold restoration may be made by burnishing a piece of pure gold 36 gauge of suitable size and shape, over the fractured area. When this is done, two or more holes for pins are drilled, being careful that the drill holes penetrate the tooth parallel to each other, and far enough from the pulp, and also from the enamel so that no harm will result from that source.

If the pin holes are not parallel the restoration will not go to place without bending the pins or distorting the gold. The pins should be fitted in and soldered to the gold with the smallest amount of solder necessary to form a strong attachment. The piece should now be returned to the tooth and re-burnished and trimmed nearly to margin of fractured area. If the pins project from the gold surface, they should be ground off, leaving a smooth surface. Another piece of gold should be added to the first and soldered and trimmed. This is repeated until the desired fullness has been obtained, after which the restoration is cemented to place and finished.

In building up a contour of this sort, the gold to be added after the first layer has been soldered may be considerably thicker than 36 gauge, which saves time and gives the same result. As an illustration of this method, I will describe a practical case treated about two years ago.

Two lower incisors and one cuspid were fractured by accident. The incisors were crowned with porcelain, but the cuspid was not so badly fractured as to require devitalization, and the force of the occlusion was too great to admit of using porcelain in building out the required contour. In this case three holes were drilled for pins. Thirty-four gauge pure gold was burnished over surface and pins soldered to place. The gold was then re-burnished and removed from tooth, after which an impression and bite were taken to aid in establishing the proper amount of fullness necessary in building the restoration. The gold was built up, a layer at a time, as has been just described, and was cemented to place at the next sitting.

The method of inlay construction which has been the most satisfactory to me, and which can be used in the greatest variety of cases, is of pure gold made in a platinum matrix. In the preparation of the cavity the usual inlay principle must be followed out. The cavity margins should be trimmed back to sound tooth structure, and the walls so shaped that a matrix may be withdrawn without becoming distorted. If the cavity is in the mesial or distal surface of a bicuspid, involving the occlusal surface, the fissure should be opened throughout its entire length. If this is done I can see no great advantage in forming a step in the occlusal surface.

In the molar teeth a step is sometimes necessary for purposes of retention, but not always, and the operator must be governed by conditions as he finds them. A gold inlay is practically a cement filling protected by a gold covering. It is the cement, not the gold, which comes into contact with the tooth structures, and if the cavity is so shaped that a cement fiillng would safely withstand the stress of occlusion, and if the gold covering is so shaped as to admit of a firm union with the cement, I can see very little advantage in cutting away sound tooth structure to get greater retention.

After the cavity is satisfactorily prepared, a matrix of 1-1000 inlay platinum is burnished to place. The matrix should be large enough to overlap the cavity considerably, and the burnishing should be especially thorough around the margins of the cavity until they are sharply defined.

The matrix is now removed from the cavity and held by the over-lapping margin in a pair of locking tweezers, and the reverse side of the matrix painted with a solution of whiting and water. The next step is to place a small piece of pure gold of about 30 gauge in the matrix and melt it with the blow-pipe flame. This is repeated until the bottom and sides have a thin covering of gold. The partially filled matrix is now dropped in full strength hydrochloric acid to remove the whiting, thoroughly rinsed and returned to the tooth for re-burnishing.

In building up the balance of the inlay the gold is not melted into the matrix, as a better result is obtained by sweating it in one piece at a time. The criticism has sometimes been made that pure gold should not be used in this work, on account of its tendency to melt up into a round ball, and thus prevent properly contouring the inlay. I have not found this to be the case, and am convinced that trouble of this sort is a result of not properly using the blow-pipe flame. The entire inlay should be heated almost to the melting point of gold before the flame is directed on the small piece of gold which is being added. When this is done it will be found that the small piece will settle into place with very little change of shape. After the inlay is built up to the proper fullness and contour, the margins should be trimmed and as much finishing as possible should be done before cementing into the tooth.

The reverse side should be roughened with a cross-cut bur. Little drill holes may be made, or grooves cut, any scheme resorted to which will give the cement greater retention, so long as the fit of the inlay is not impaired. The final finishing should be done in the mouth.

In mixing the cement for setting the inlay, it must be bourne in mind that the inlay fits the cavity very closely, and consequently the cement must be thin, or there will be trouble in getting the inlay to place. The cement should be thoroughly spatulated, and both the cavity and cavity side of the inlay should be covered with cement before inserting the inlay.

In addition to the filling of cavities, inlays of this description may he safely used as one of the abutments for short bridges; but in this case the retention should be supplemented by the addition of one or more pins, projecting from the under side of the inlay into pits drilled into the floor of the cavity.

The hollow gold inlay is used oftentimes in cases of badly broken down molar and bicuspid teeth. One method of construction is as follows: After the cavity is prepared, a matrix of pure gold 36 gauge is fitted to the cavity. This may be done by taking an impression of the cavity in modeling compound. A cement or amalgam model of the tooth can be made from this impression, and the gold matrix formed in this model. A hole is cut in the bottom of the matrix, after which it is placed in the cavity and burnished until the fit is perfect. The matrix should be removed from the cavity and annealed frequently during the burnishing process.

When the matrix is satisfactorily fitted and in position in the cavity, it should be filled with soft dentallac, and the patient is instructed to bite. This dentallac is intended to serve as a core over which the remaining portion of the inlay is to be swedged, and should be so shaped as to bring out the correct fullness and contour, minus the thickness of gold which is to serve as the occlusal and approximal surfaces. The matrix is now removed from the cavity with the dentallac core in position. The reverse side of the matrix is filled with cement, after first being oiled to prevent the cement from adhering too firmly. This cement is to serve as a foundation to keep the matrix from becoming distorted during the swedging process, and there should be bulk enough of the cement to give it strength. Next cut a piece of pure gold, 34 gauge, and large enough to entirely cover the dentallac core, and lap oyer the cavity margins slightly. This piece of gold is to be formed over the core and several slits may be cut in it from the edge, almost to the center, so that it will more easily conform to the shape of the core. After forming this as well as possible by bending the edges and burnishing, it is swedged in a Place, or other similar swedger. After removing from the swedger the core is taken from the matrix, which is carefully loosened from the cement foundation, and the two parts of the inlay united with solder. The occlusal surface is thickened by adding solder through the hole in the bottom of the matrix. The inlay is cemented to position in the usual way, the final finishing being done in the mouth.

The cast gold inlay is the most recent development in inlay construction, and promises to be the best, especially for large cavities in molar and bicuspid teeth. So much has been written on this subject of late that we all have a general idea of the way the cast inlay is made. but as I have not done any of the work myself, I shall not attempt to discuss it.

In conclusion, I would say that we need the gold inlay, because of the imperfections and limitations of the materials upon which we have so long depended.

The gold inlay has proved beneficial to the operator and also to his patient, for it has lessened the strain and exhaustion of dental operations for both. It has enabled me to insert durable and sightly fillings iu the molar teeth and by its use I avoid resorting to the gold crown in many cases which were formerly treated in that way.

It has provided me a safe and sanitary means of obtaining abutments for short bridges, and it has reduced the necessity for the unsightly and uncertain amalgam filling which I have so often been compelled to insert.

### DISCUSSION.

Dr. E. B. Spalding: What I have to say will be in support of inlays as a means of filling teeth, and not of the technique of the gold inlay. It has been my privilege in the last few years to see many very beautiful gold inlays inserted, and though a porcelain worker, I am intensely enthusiastic for the gold inlay, and realize its field of usefulness. While my experience has been confined to porcelain inlays, I realize that it has limitations, but do not regret that a number of years ago I took the porcelain inlay seriously, and believed that it must take a large part of our work. The limitations of the porcelain inlay are largely met in the gold inlay. For those places in the mouth which receive the most stress the cast gold inlay can be substituted to advantage, especially the gold inlay.

I am very much pleased with Dr. Hopkins and his paper, because he has treated his subject so seriously, and believe he has good reason to be enthusiastic. He is undoubtedly using the inlay as much as any one else in this city, and what he has said is the results of his own experience: and you know that we have more confidence in the man who is doing things than we have in the man who is only preaching.

Two years ago, at the time of the First District Society Clinic here, there were two prominent dentists of Chicago here to take part in our program, and one of them is an inlay enthusiast. He came to the office to see me, and I was busy at the time, but invited him into the operating room. He looked around a moment, and said, pointing to the gold annealer:

"And what is that?"

I answered, "That is a gold annealer."

He said, "Have you any use for that?"

"Certainly I have."

He said, "Why don't you practice what you preach?"

I said, "I thought I did."

He said, "You do not mean to say that you are using gold?"

"Most certainly I am, and hope to use it for a good while."

"You are not consistent; I am ashamed of you."

But within the last week I have learned that this same man has purchased a Taggart gold inlay casting outfit, and has asked that he may have the privilege of demonstrating it to a number of dentists the last of this month. I think he also is a little inconsistent.

The essayist asks the question, "Why is the gold inlay needed, and what advantages has it to offer not possessed by long established methods?" He has answered these questions very satisfactorily, I think, but I would like to emphasize a little. Our patients come to us to have their teeth made comfortable; to have them preserved, and to have them made useful and sightly. Now, if we dam them with a rubber dam, and mallet them for an hour or two, inserting large gold fillings, we certainly are not making them comfortable for the time at least. And then, how many large gold fillings are most uncomfortable from thermal shocks. They cause the death of the pulps frequently, and for days and months after a gold filling is inserted it is certainly very unpleasant for the patient to take a glass of cold water. I

believe that many of those fillings that are constantly shocking the pulps, eventually cause the death of the pulps. Are we making our patients comfortable by doing operations in that manner? We could use inlays which are inserted very much more easily, and are quite as durable, and protect the tooth from recurrent caries fully as well. I should like to know what percentage of inlays have caused the death of pulps. Have the members of this society found it necessary to remove inlays to treat putrescent pulps, or to drill into pulps to treat teeth that have had inlays inserted, especially when the pulps were not exposed and infected when the inlay was inserted? That layer of cement which the inlay has between the metal or the porcelain, whichever it may be, and the tooth substance, is truly a comfort to the tooth and to the patient, and a blessing to the operator.

Dr. Hopkins mentioned contouring the inlay. In inserting an inlay it is nearly always necessary to make considerable separation, and because of that we are able to shape an inlay to the natural contour of the tooth. Are we as careful when we are constructing a gold or an alloy filling? I am afraid we are not; at least I know I was not when I was using more gold and alloy than I am at present. The polishing of our fillings often destroys the contour which we may have built, unless we use a separator which of course is in the way in finishing the filling.

Another point which Dr. Hopkins brought out, and which I would like to emphasize, is the condition of the gum tissue about an inlay, especially a large restoration of a molar instead of a gold crown. Any crown which has a band jeopardizes the health of the gum tissue, as it tends to destroy what some one has termed the natural water-shed of the gum. The band crown necessarily has a lap joint, and, though that lap is made as perfectly as possible, does not compare with the flush joint of an inlay, and the band gold crowns are the causes of innumerable cases of pyorrhoea. The gold inlay has one advantage over the porcelain inlay at the joint, because the porcelain inlay must remain as it is made, while the gold inlay can be finished and polished in the mouth, and the edges can be made flush and finished to a perfectly tight joint. Where the porcelain inlay is used there is always a small amount of space, no matter how well made the inlay is. I would not have you understand that I condemn the porcelain inlay, but this is an advantage which the gold inlay has over the porcelain, and especially with the cast gold inlay, where only pure gold is used without the platinum matrix. I understand from those who have made the cast gold inlay, and in fact have seen cases where the joint is very much tighter and better on account of the ability to finish the gold closest to the cavity margins. This affords protection to the enamel margins, and is another thing in favor of the gold inlay, especially on cuspids, molars and bicuspids, where there is a great deal of stress. The edges of a porcelain inlay are liable to chip and expose the enamel so it will chip. It does not support the enamel of the tooth as does the gold. Of course the gold inlay cannot compete with the porcelain from the aesthetic point.

The essayist did not say anything with reference to the tendency of inlays to become uncemented; possibly he does not have them do so. Unfortunately, I have them now and then, and I believe the two principal reasons, as I believe most every one will agree, is the faulty cavity preparation or faulty cementation. I do not believe that saucer-shaped cavities, with no form or depth, can be filled successfully with either porcelain or gold inlays. A few years ago it was advocated that if you put enough pressure on a porcelain inlay it should be retained in any shaped cavity; but I now believe that it is absolutely necessary to have a certain amount of form and seating retention for all fillings. The cavities with which I have had the most trouble in cementing inlays are cervical cavities near the gum line, where it has been next to impossible to have that cavity absolutely dry when the cement is introduced. The cavity walls may appear perfectly dry, but if there is a bit of moisture under the gum the cement is liable to cleave from the walls of the cavity. I liken it to plaster which has set on a glass slab; when it is dry it is exceedingly hard to detach from the slab, but let us put a little moisture on one side and it soon loosens. I believe that cement is loosened from the cavity in the same way, and that this moisture infiltrates under the cement, as it seems to be in those cavities where it is very difficult to keep the margin perfectly dry when the cement is introduced that we have the most trouble. I have been told that Dr. Ames is advocating moistening the cavity with pure water before the cement is introduced. I would have to be shown that he has a much more successful method than having a dry cavity before I can believe it.

The inlay is not as wearing upon the nervous system of the dentist or of the patient either as other operations which require continuous working over a patient. I think that we ought to consider this fact carefully, because we know of several instances of dentists who are breaking down their health by too conscientious continuous work over their patients.

Several years ago I met Dr. E. C. Moore on the street, and he was telling me about the construction of a plate. I had not been out of college long then, and I said: "Dr. Moore, I dislike doing plate work; I do not care anything about it." He floored me by saying, "Well, the reason you don't care anything about it is because you don't know anything about it," which I knew was only too true. Then he went on to say that when a dentist had practiced as long as he had, he would be very glad to sit down on a chair in his laboratory and work at the bench in preference to working over patients. I believe he was right, and any process which will give us a rest from the continuous work over a nervous patient should be beneficial to ourselves.

Dr. J. W. House, Grand Rapids: Mr. President, and members of the First District Dental Society: I do not believe that I can add anything to what has been said, except to indorse the essayist's methods of procedure. I appreciate the privilege of being here. I have always felt that you had to come to Grand Rapids to be shown things, but I assure you that when I see this large number of representative Detroit dentists, and note the close and eager attention given to this important subject, that I feel somewhat different. I know that it will stimulate the profession in Grand Rapids to learn from me of the large number that you have here tonight, and of the interest and enthusiasm of your meeting, and I sincerely trust that we may have at our next meeting as good a representation as you have here. I can assure you that if any of you come to Grand Rapids we shall try to make it as interesting as this meeting seems destined to be.

DR. M. L. WARD: I, for one, feel very grateful to Dr. Hopkins, as we all understand that his paper was to have been presented several months later, but owing to the failure of the essayist for this evening, he obligingly prepared on short notice for this meeting.

I am sure the essayist is a good, thorough workman, and I am also very much pleased with the comprehensive way in which he has presented the subject. Almost everyone nowadays gets so infatuated with his hobby that when he presents it in public he thinks he must say all the good things he can think of, and never tell any of the unfavorable things. Now you notice that he has not done that.

One of the most valuable features of the gold inlay is that it does away with so much alloy work. It seems as though alloy work has been cut out 75 per cent in the better practices since we began to use gold inlays. There is one thing about the gold inlay in my hands that is decidedly in its favor, and that is: For frail teeth in which you are troubled continually in getting retention form, and in which you can hardly get any form in any way, pits or undercuts for retention, you can do with the inlay what you cannot do with alloy or gold either.

I feel that inlays are not as strong as the entire cement fillings, as no inlay cement is as strong as the cement used for a filling, as you cannot use inlay cement at its best consistence. If you mix them both to the same consistency, the inlay cement attachment is weaker. There is always more acid in the inlay cement than there should be for a good filling. This leads me to think that where a cement filling will hold, an inlay may not. I have been using almost entirely the ordinary cement. I have always thought the cement must be faulty when my inlays came out. Of course there may be a great deal more strain on these fillings than on any cement filling. I have been using the ordinary cements, and believe that the idea that these cements are too coarse is a farce. I have never seen an inlay yet that fit so tight that a reasonably well mixed cement could not be used to cement it to place. The ordinary cement in my hands has been more successful than inlay cement.

The point made in connection with the gold inlay that makes it a good filling is that you finish it in the mouth, and therefore have no projecting margins, is a valuable one. It is very difficult to detect any margin at all in a properly constructed and fitted gold inlay. I have had, up to the first of last week, very questionable results with the cast gold inlay, and I have had some experiences which possibly may be interesting to some of you. I began casting with the Kenyon outfit, but soon found it was not designed as perfectly as it ought to be, and changed it for the Custer apparatus. I tried to use the Custer outfit, and had success sometimes and sometimes not. On New Year's day I spoiled two out of three practical cases; but I had made up my mind that it was possible to do good gold casting with this machine. I found out first of all that the pressure used must be somewhere near uniform to get successively uniform results. The air pressure in our laboratory is secured by automatic electric pumps, giving 40 pounds one time, and 20 pounds, perhaps, at the next. I also found that when I had a green or fairly soft mould, and turned that pressure of 40 pounds on it, it will blow the bottom out of the mould or blow the gold into the plaster; at any rate, it would be warped so that the casting would not fit the cavity. I found that Dr. Custer used a quart tin can containing air under 20 pounds of pressure only, and there was one gush, and that was all the air he had. I got a regulator and put it on our air, and since that time have been using 21 or 22 pounds.

I found out another thing: That little sprue wire that you stick into the wax filling was pointed, and consequently would stick into the wax a little one time, and a little further the next time, giving no regular size to the sprue hole or casting duct. I got some No. 14 wire, and have it perfectly straight all the way, except I have turned a little ring on the end that you stick into the wax to make it hold better. Every time I use that wire it goes in just as far each time, and the hole is always the same size; and now with uniform air pressure, I have good success. It does seem as if this thing is worked up right, and the casting machines can be had for a reasonable price, they must be in the office of every dentist. If my experience may be of any value, I am glad to have related it to you.

Dr. G. C. Bowles: I have done quite a little inlay work, but have little to add to the technique, as I think Dr. Hopkins has covered the field pretty well. I followed the Doctor in the preparation of his paper somewhat, and I know of the care he took in every step and phase of that paper. His paper differs, as Dr. Spalding pointed out, from every other paper we have heard read, in that every step that he described is a step that he has followed in his practice. Albertus Hubbard said of Thomas Edison on one occasion, that it was a good thing that Edison was not an educated man. He said in Harvard they were discussing at one time a scientifie problem, and it was stated that a light could not be made in a vacuum, and while they were discussing it so wisely, Edison went to work and made one. It is demonstration that we want, not theory. Theories are good in their place, but we want practical facts.

It may be that Dr. Hopkins is more careful in the preparation of his cavity seat than many of us. I told him, when he read the paper to me, it had become a sort of a religious tenant with me to make a step in every case I could, and if I was called to put in a filling without a step, I had a twinge of conscience. He said he did not do that in many cases.

The paper does not give much chance for criticism; one cannot object to the statements very well. About Dr. Ames and the water cement: I read Dr. Ames' article on that subject, and I rather suspect that Dr. Spalding and Dr. Mueller have Dr. Reeves' phase of that thing. As I remember it, Dr. Ames said, dry a cavity, and moisten it with the liquid of cement, and not water. I think possibly Dr. Reeves has modified it.

The inlay filling does not solve the problem, necessarily; we can make bad inlays with a cement which will make a good filling; I know that, because I have made a whole lot of them. There have been about as many as the alloy and gold fillings that I have made. I really think for an average operator, I get better results with gold inlays than I ever did with gold fillings, and they last better, and are going to give better results.

Dr. A. L. Le Gro: As some of you know, I have been interested in gold inlay work for a number of years—five or six—and although the subject has been pretty thoroughly covered, there are some things I should like to speak of.

Inlay work, we have been told, is as old as the art of dentistry itself, but it is for us to practice only the new adaptations of the method. I contend, as I have always, that the sweated gold inlay is the ideal inlay. I have tried out three or four different machines in this cast inlay business in the last five months. I finally got a Custer electric, and my experience has been very similar to Dr. Wards.' I had the same troubles that he encountered. I would get some beautiful results, and all of a sudden at a most inauspicious moment, the machine would disappoint me. I talked with Dr. Custer at Minneapolis, and he said that while he recognized the very good qualities of the Taggart machine, he thought Taggart made a great mistake in not fusing the gold by the electric arc. It is done very quickly, and is an ideal way.

When I first started doing porcelain inlay work, I would get a pretty fair inlay occasionally, and when I put it in the tooth it looked very nice, and the margins, under a magnifying glass were beautiful; but when I mixed the cement according to instructions, and put it into the cavity and set the inlay, I would find there was a considerable line of cement all around the inlay. I was talking with Dr. Taggart this summer about that and he has given, I think, about the best theory that I have ever heard along the line of adaptation of cement in the inlay cavity. He claims that when we put the inlay into the cavity and force it in under pressure as we would do it in the beginning, that the cement granules are crowded upon one another, and are not allowed to shift into their position relative to one another, and the result is the inlay is crowded out to some extent, in fact so that it is perceptible to the eye. If you stick a good sized post into sand and try to force it down into the sand, you cannot do it, but if you wriggle it sideways you can work it down. I have tried this method with the inlay to some extent, and find that it has some virtue. There is no question but what alternate jolting of one side of the filling and then the other to put it thoroughly into place will more evenly distribute these little granules of cement.

The essayist spoke about cement sticking to gold. I have tried a number of experiments in regard to three different cements, and have found that they all behave similarly on similar golds. For instance, cement will stick better on pure gold than any other form of gold or precious metal used. Just why this is I am unable to say, but it is a fact, just the same.

Dr. Spalding advanced the theory that a cast gold inlay made of pure gold would form a better joint than a gold inlay with matrix. While I am not able to cast any reflections on the cast gold inlay, because where it is successful I think it is the most perfect filling made; but the gold inlay made in a platinum matrix 1-1000. or even 1-800, in burnishing the gold to the beveled margin, and we can always have beveled margins in gold inlays, the bottom of the gold becomes pushed by the burnisher, pushes the surface of the matrix so that the matrix can be just as well adapted to the border as the gold inlay. In regard to gold inlay work, I think it is the duty of every dentist to take up gold inlay work. Dr. Spalding very nicely illustrated the fact of his experience with Dr. Moore in regard to gold. The reason some of us condemn gold inlays is because we do not know enough about them. It is up to us to go into our laboratories and make 12 or 15 models. Don't go to your patients and think you are going to stick in a gold inlay just as quick as somebody shows you the technique. Even when you have made ten or twelve successful inlays in your laboratory, you are liable to have failures with your patients. So it behooves us all to take up this work, because inlay work certainly has a place in dentistry.

DR. T. R. BUTTRICK: I would like to add one thing along the line of cementation. I do not know whether Dr. Reeves has modified his ideas about moistening the cavity with water or not, but I had the pleasure of being over to Chicago a year ago, and at that time he moistened the cavity with a little of the finely mixed cement, then mixed it in a little thicker consistency, and set his inlay. My idea is that if you dessicate the teeth and get it so dry that it absorbs a certain amount of the moisture from the mix, you would get a good set; you cannot get your inlay in place. I have tried this in teeth that I have had to build up with cement, where there was deep undercutting made and the teeth get thoroughly dry, and find the dry tooth will take the moisture away from the cement mix so rapidly that before you get the inlay home it will set. You have got to wet your cavity or it will take the moisture away so that it will set before you get it home.

DR. HOPKINS: Sometimes gold inlays are finished too soon after cementing them in place. Force exerted against the inlay when cement is not thoroughly hardened will cause it to become partially dislodged, and that may account for some of the failures operators have in regard to inlay work.

I think my experience has been similar to Dr. Ward's in regard to the cement. I think that the ordinary cement you use has been more successful than the special cement used in setting inlays, especially where there has been satisfactory occlusion and you require a strong cement. Sometimes when the inlays are in such a position that they could not become dislodged in any way, it does not make any difference.

The platinum matrix method as compared to the cast method: It would seem to me that for small cavities you would get your inlays done much more rapidly with the platinum matrix method, and have the margins fully as good. It seems to me that you are complicating matters by attempting to cast an inlay for small cavities.

